# Hair Tourniquet Syndrome Involving the Uvula Secondary to an Airway Foreign Body

**DOI:** 10.7759/cureus.53656

**Published:** 2024-02-05

**Authors:** Theresa A Schneider, Jatin Ahluwalia, Ani Mnatsakanian, Michael Haupert

**Affiliations:** 1 Otolaryngology - Head and Neck Surgery, Ascension Macomb Oakland Hospital, Warren, USA; 2 Pediatric Otolaryngology, Beaumont Royal Oak Hospital, Royal Oak, USA

**Keywords:** upper airway, constriction, airway foreign body, uvula, hair tourniquet syndrome

## Abstract

Hair tourniquet syndrome is a rare condition that can cause ischemia and necrosis secondary to hair fibers constricting a patient's appendages. Typically, the syndrome affects patients aged two to six months. Hair tourniquet syndrome often involves the toes, fingers, or genitalia, and it has been rarely reported to have oropharyngeal manifestations. Accurate and timely treatment of this syndrome is imperative to save the involved appendage. We discuss a case of a six-month-old female who presented to the emergency room (ER) with increased agitation and was found to have hair tourniquet syndrome of the uvula, requiring the removal of the foreign body in the operating room (OR).

## Introduction

Hair tourniquet syndrome is defined as the constriction of blood flow to an appendage, typically by a hair or textile fiber. The appendages most commonly affected include toes, fingers, and genitalia, but the condition can involve other structures as well [1]. Although hair tourniquet syndrome does not commonly have oropharyngeal manifestations, there have been rare reports of uvula and tooth involvement. Typically, hair tourniquet syndrome presents in patients aged two months to six months and often corresponds to the telogen effluvium phase of postpartum maternal hair loss. The mechanism of injury likely begins with the restriction of lymphatic drainage, further progressing to lymphedema and venous outflow obstruction resulting in ischemic injury [2]. A prompt diagnosis is critical to prevent further complications, such as strangulation, necrosis, and autoamputation of the involved appendage. Most cases are accidental, with a small proportion attributed to nonaccidental injury. While the treatment commonly involves removal by direct inspection, removal under surgical exploration may be required if the patient is not cooperative or visualization of the fiber is lost due to incomplete removal or fiber cutting deeper into the skin or mucosa [3].

Upper airway foreign bodies are most commonly found in patients under three years of age and are often diagnosed late unless the patient was directly witnessed ingesting the object. The condition is often misdiagnosed as croup, upper airway reactive disease, or laryngomalacia initially. Foreign bodies of the upper airway often produce symptoms of obstruction, such as stridor, wheezing, chest retractions, and/or persistent cough, and should be suspected in patients who do not respond clinically to treatment for upper airway infection or upper airway reactive disease [4]. The diagnosis of an airway foreign body can be aided by fiberoptic bronchoscopy or a CT scan [5]. We describe a rare case of hair tourniquet syndrome of the uvula secondary to an upper airway foreign body.

## Case presentation

The patient was a six-month-old female with no significant medical history who presented to a pediatric emergency room (ER) as a transfer from another facility. She had been initially seen at the outside facility with increased agitation. She was noted to have a small band of hair wrapped around the uvula and extending out of the mouth. The patient’s parents had not directly witnessed the patient placing any foreign body into her mouth. The parents were unsure how long the foreign body had been in place. At the outside facility, attempts had been made to remove the foreign body using direct removal. The uvula had been noted to swell progressively and engorge with increased manipulation.

**Figure 1:**
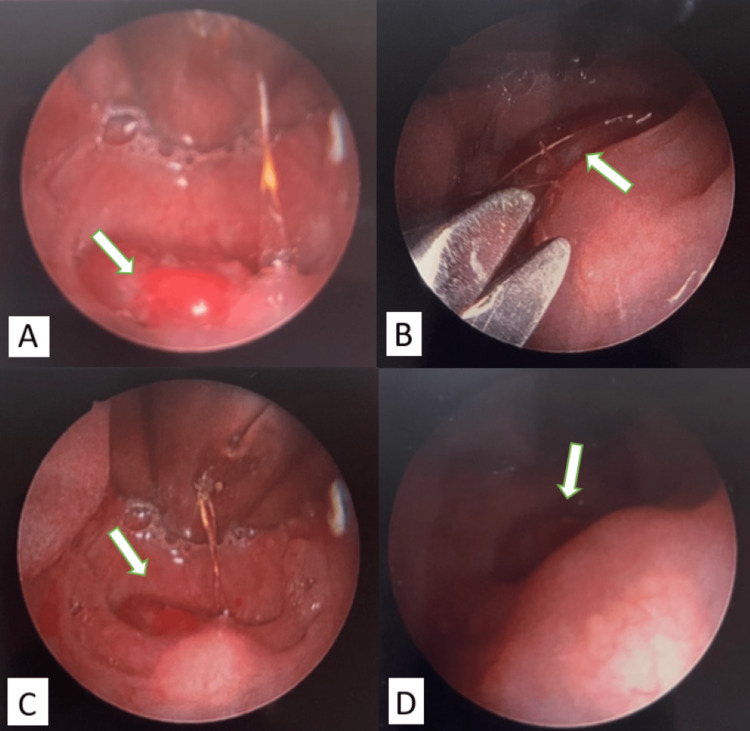
Images of hair tourniquet on the uvula and the removal of the foreign body A. Endoscopic Images of hair tourniquet on the uvula. B. Surgical removal of hair fiber with iris scissors. C. Uvula immediately after removal; engorgement and edema of the uvula can still be seen. D. Uvula five minutes after the removal; edema improved significantly

Upon presentation to the pediatric ER, the patient was hemodynamically stable and in no respiratory distress. Her vitals were as follows - blood pressure 105/81 mmHg, heart rate: 108 beats per minute, oxygen saturation: 100%, and respiratory rate: 28 breaths per minute. She did not have stridor, stertor, drooling, or increased work of breathing upon examination. Initially, the ER team did attempt bedside removal. Again, there was concern for possibly worsening the uvular strangulation and edema, and otolaryngology (ENT) was consulted. Upon ENT evaluation, the foreign body was again visualized (Figure 1). The decision was made to induce conscious sedation and attempt the removal of the object. The patient was laid in a supine position and placed on a monitor. A nasal cannula was also placed. Intravenous ketamine was used for conscious sedation. Using a size 1 Miller blade, the tongue was displaced and the foreign body was visualized. Iris scissors were used to try to release the foreign body. However, these attempts were unsuccessful. Hence, a decision was made to proceed to the operating room (OR) for direct laryngoscopy, bronchoscopy, and foreign body removal. 

In the OR, general anesthesia was initiated and the patient was endotracheally intubated. A McIvor mouth gag was placed and an endoscope was used to visualize the foreign body. Using iris scissors and Debakey forceps, the foreign body was carefully cut and removed from the uvula and extracted from the oropharynx (Figure 1). There was no damage noted to the uvula post-extraction, although edema and engorgement of the uvula were still present. The hypopharynx, supraglottis, glottis, and trachea were then examined using an endoscope to confirm the absence of any additional foreign body. The patient was then successfully extubated and taken to recovery in stable condition. She was discharged home following the procedure.

## Discussion

We presented a rare case of hair tourniquet syndrome with uvular involvement due to an upper airway foreign body. There have been a few reports of hair tourniquet syndrome with involvement of the upper airway, including two reports of uvular involvement and one case involving a tooth, with the ages of these three patients ranging from seven months to two years [2,6,7]. Situations involving severe hair loss from a caregiver, such as postpartum telogen effluvium or chemotherapy-induced anagen effluvium, put young patients at risk. Telogen effluvium peaks at two to six months postpartum, which corresponds to the most common age among the patient population involved [8]. Using old clothing or mittens can also result in textile fiber-induced tourniquet syndrome [9].

Maintaining a high index of suspicion is critical to prevent complications of hair tourniquet syndrome. These complications include strangulation, necrosis, and autoamputation of the involved appendage [3]. Irritability is Often the only presenting symptom, especially in the early stage of the presentation [10]. Treatment should involve using the least invasive method, which is typically mechanical removal using scissors or similar instruments under direct visualization. Direct visualization is needed for removal, and it requires a well-lit room, preferably with magnification devices. In some cases, depilatory creams have been used to aid removal. If necessary, pain control and light sedation, such as ketamine, can be used to increase patient cooperation [11].

In the setting of continued difficulty in the removal of the fiber, such as in our case, moving the patient to the OR for examination under anesthesia may be necessary. We were able to remove the constricting hair fiber successfully in this case under general anesthesia. However, if the difficulty persists, a longitudinal incision can be made perpendicular to the constricting fiber with careful consideration of nearby neurovascular structures. In some of these difficult cases, formal surgery with deep incision down to the bone may be required to obtain definitive treatment of the strangulation [11]. This may be necessary in cases with severe inflammation and deep displacement of the fiber. 

There are a few complications associated with the removal of hair or textile fiber in a patient with hair tourniquet syndrome. Hair is a thin fiber that can be difficult to visualize, especially if there is a local reaction with swelling, which can further impair visualization. Hair expands when wet, but constricts as it dries, leading to further constriction of the involved appendage [12]. Due to constriction and delayed recognition of hair tourniquet syndrome, there can be reepithelialization over the fiber, resulting in additional difficulty in establishing the diagnosis [10].

## Conclusions

Hair tourniquet syndrome is most commonly seen in the pediatric population. Extreme caution should be exercised when encountering patients presenting with hair tourniquet syndrome of the upper airway structures, as this can lead to more significant complications, such as edema and strangulation, and may involve airway foreign bodies. In less obvious presentations, additional diagnostic tools such as flexible scopes may be used to aid the diagnosis. Prompt diagnosis and treatment are critical to salvage any involved appendages in hair tourniquet syndrome.

## References

[1] Claudet I, Pasian N, Maréchal C, Salanne S, Debuisson C, Grouteau E (2010). Hair-thread tourniquet syndrome (Article in French). Arch Pediatr.

[2] Dey R, Zameer MM, Vinay C, Rao S (2022). Hair tourniquet of the uvula. J Indian Assoc Pediatr Surg.

[3] Mackey S, Hettiaratchy S, Dickinson J (2005). Hair-tourniquet syndrome--multiple toes and bilaterality. Eur J Emerg Med.

[4] Kamath MP, Murthy PS, Hazarika P (1999). A case of foreign body in the subglottic region. Indian J Otolaryngol Head Neck Surg.

[5] Yan S, Jiang P, Chen G, Chen Y, Pan H, Li L, Zeng N (2022). Characteristics and treatment of pediatric tracheobronchial foreign bodies: a retrospective analysis of 715 cases. Med Sci Monit.

[6] McNeal RM, Cruickshank JC (1987). Strangulation of the uvula by hair wrapping. Clin Pediatr (Phila).

[7] Flores JR (2014). Hair tourniquet syndrome in the dental patient. Anesth Prog.

[8] Strahlman RS (2003). Toe tourniquet syndrome in association with maternal hair loss. Pediatrics.

[9] Serour F, Gorenstein A (2003). Treatment of the toe tourniquet syndrome in infants. Pediatr Surg Int.

[10] García-Mata S, Hidalgo-Ovejero A (2009). Hair tourniquet syndrome of the toe: report of 2 new cases. J Pediatr Orthop.

[11] Sivathasan N, Vijayarajan L (2012). Hair-thread tourniquet syndrome: a case report and literature review. Case Rep Med.

[12] Alpert JJ, Filler R, Glaser HH (1965). Strangulation of an appendage by hair wrapping. N Engl J Med.

